# Relationship between Proinflammatory and Antioxidant Proteins with the Severity of Cardiovascular Disease in Type 2 Diabetes Mellitus

**DOI:** 10.3390/ijms16059469

**Published:** 2015-04-27

**Authors:** Beatriz García-Fontana, Sonia Morales-Santana, Victoria Longobardo, Rebeca Reyes-García, Pedro Rozas-Moreno, José Antonio García-Salcedo, Manuel Muñoz-Torres

**Affiliations:** 1 Bone Metabolic Unit, Endocrinology Division (RETICEF), Instituto de Investigación Biosanitaria (Ibs) Granada, University Hospital San Cecilio, Granada 18012, Spain; E-Mails: bgfontana@fibao.es (B.G.-F.); sonia.morales.exts@juntadeandalucia.es (S.M.-S.); rebeca.reyes.garcia@gmail.com (R.R.-G.); 2 Proteomic Research Service, Instituto de Investigación Biosanitaria (Ibs) Granada, University Hospital San Cecilio, Granada 18012, Spain; 3 Proteomic Research Service, Institute of Parasitology and Biomedicine “López Neyra” (C.S.I.C.), Granada 18016, Spain; E-Mail: vlongo@ipb.csic.es; 4 Endocrinology Division, Ciudad Real General Hospital, Ciudad Real 13005, Spain; E-Mail: pedrorozasm@yahoo.es; 5 Infectious Diseases Unit, Instituto de Investigación Biosanitaria (Ibs) Granada, University Hospital San Cecilio, Av. Dr. Olóriz 16, Granada 18012, Spain; 6 Endocrinology Unit, University Hospital San Cecilio, Av. Dr. Olóriz 16, Granada 18012, Spain

**Keywords:** cardiovascular disease, type 2 diabetes mellitus, proinflammation, oxidative stress, proteomic

## Abstract

Type 2 diabetes mellitus patients are at significant risk of cardiovascular disease, however, the pathophysiology of these complications is complex and incompletely known in this population. The aim of this study was to compare the serum proteome of patients with type 2 diabetes mellitus presenting or not presenting cardiovascular disease with non-diabetic subjects to find essential proteins related to these cardiovascular complications. This cross-sectional study compares the serum proteome by a combination of protein depletion with 2D-DIGE (2-dimension Difference Gel Electrophoresis) methodology. The proteins differentially expressed were identified by MALDI TOF/TOF (Matrix-assisted laser desorption/ionization and Time-Of-Flight ion detector) or LC-MS/MS (Liquid Chromatography coupled to Mass-Mass Spectrometry). Type 2 diabetes mellitus patients with cardiovascular disease showed higher expression of plasma retinol binding protein and glutathione peroxidase-3 compared to those without cardiovascular disease and non-diabetic controls. These results show that proteins related to the inflammatory and redox state appear to play an important role in the pathogenesis of the cardiovascular disease in the type 2 diabetes mellitus patients.

## 1. Introduction

In developed countries, type 2 diabetes mellitus (T2DM) represents a major public health problem mainly by its relationship with different cardiovascular diseases (CVD) [1]. Long-term hyperglycemia results in the synthesis of a number of molecules like advanced glycation end products (AGEs), advanced oxidation protein products (AOPPs), and low-density lipoprotein susceptibility to oxidation (oxLDL). Besides chronic hyperglycemia, other factors such as insulin resistance, dyslipidemia and states of inflammation and oxidation are related to vascular injury in diabetes through several underlying processes [2]. However, only some diabetic patients develop cardiovascular disease while others do not develop these complications despite having the same common risk factors. The etiologic pathway linking impaired glucose tolerance and cardiovascular disease remains to be clarified. Many factors, including genetic components may be involved and not all are well established. Currently, multiple areas of research are open to explain this complex phenomenon [3] and the precise role of the different disturbed metabolic pathways is not well established. In this context, the identification of new molecules that take part in the development of these vascular complications in T2DM may be of great importance for improving the outcomes of this population or to design new therapeutic targets. Thus, the underlying factors that may lead to the development of CVD require further in-depth research. The proteomic analysis is a hypothesis-free approach integrating genetic and epigenetic influences by examining the protein expression profiles and is not limited by the above knowledge.

Human serum is rich in potential biomarkers reflecting the pathophysiological state of the human body and related to the impaired metabolic pathways present in different disorders. In this way, it is able to facilitate the early detection of many diseases. However, finding biomarkers in serum is difficult due to the interference of the main proteins that provide a significant background covering low abundance proteins [4].

To overcome this problem, serum depletion is essential for removing the major proteins and to improve the sensitivity of detection methods for these minor proteins.

The main objective of this cross-sectional study was to compare the serum proteome of the T2DM patients with and without CVD and non-diabetic controls to find protein differences in the protein profile expression of the study groups. Candidate proteins were identified by MALDI TOF/TOF or LC-MS/MS in order to identify essential proteins related to severe cardiovascular disease in patients with T2DM.

Here we report that proteins involved in inflammatory and redox state, such as retinol binding protein (RBP4) and glutathione peroxidase 3 (GPx-3) have increased levels in the serum of T2DM patients with severe CVD compared to those without CVD and non-diabetic controls. These differentially expressed proteins could be potential markers used for diagnosis or prognosis of CVD.

## 2. Results

### 2.1. Baseline Characteristics of the Study Population

The clinical and demographic characteristics of the study groups (Control, T2DM + CVD, T2DM − CVD) are summarized in Table 1. All groups were comparable in anthropometric and biochemical parameters except for those associated with T2DM, CVD and the related medication. As expected, fasting glucose and glycated haemoglobin (HbA1c) were significantly higher in the T2DM patients compared to the controls. The T2DM group presenting CVD showed a longer duration of diabetes compared to the diabetic group without CVD. Moreover, there was significantly higher percentage of patients with abnormal intima-media thickness (IMT) and aortic calcifications in T2DM patients with CVD compared to the control group.

**Table 1:** Anthropometric and biochemical parameters of the study population.

Group	Control (*n* = 6)	T2DM + CVD (*n* = 6)	T2DM − CVD (*n* = 6)
Age (years)	56 ± 4	58 ± 4	49 ± 11
**Measurements**			
BMI (kg/cm^2^)	28.9 ± 3.8	29.2 ± 3.2	25.6 ± 4.2
Fasting glucose (mg/dL)	88.1 ± 7.8	202.6 ± 64.4 ^a,*^	156.6 ± 64.9 ^a,*^
HbA_1C_ (%)	4.5 ± 0.2	9.6 ± 2.9 ^a,*^	7.8 ± 2.4 ^a,*^
sBlood pressure (mm·Hg)	133.3 ± 15.1	123.3 ± 34.4	121.6 ± 27.1
dBlood pressure (mm·Hg)	83.3 ± 13.6	66.6 ± 13.6	77.5 ± 7.5
LDL cholesterol (mg/dL)	129 ± 21	83 ± 59	127 ± 20
HDL cholesterol (mg/dL)	52 ± 10	54 ± 24	47 ± 10
TGs (mg/dL)	150 ± 70	169 ± 151	177 ± 155
IMT (mm)	0.6 ± 0.1	0.9 ± 0.1 ^a,*^	0.77 ± 0.2
Creatinine (mg/dL)	0.9 ± 0.2	1.0 ± 0.2	0.9 ± 0.1
GFR (mL/min)	90.5 ± 16.6	84.8 ± 13.7	97.3 ± 11.9
Homocysteine (µmol/dL)	13.1 ± 3.8	9.5 ± 2.5	10.3 ± 3.8
**Medical History**			
Duration of diabetes (years)	–	15 ± 8 ^a,**^	10 ± 5 ^a,**^
Cerebrovascular disease (%)	–	(1/6) 16.7%	–
Peripheral artery disease (%)	–	(2/6) 33.3% ^a,*,b,*^	– ^b,*^
Coronary heart disease (%)	–	(5/6) 83.3% ^a,**,b,**^	– ^b,**^
Carotid plaques (%)	–	33.3 ^a,*^	16.7
Aortic calcifications (%)	–	40 ^a,*^	16.7
Active smokers (%)	66.7	83.3	66.7
Sedentarism (%)	50	50	33.3
Alcohol (%)	16.7	50 ^a,*^	33.3
**Current medication**			
Statins (%)	16.7	83.3 ^a,*,b,*^	33.3 ^b,*^
Oral antidiabetic drugs (%)	–	100 ^a,**^	83.3 ^a,**^
Insulin (%)	–	50 ^a,*^	33.3

BMI: Body mass index; HbA_1C_: Glycated haemoglobin; s: Systolic; d: Diastolic; LDL: Low density lipoproteins; HDL: High density lipoproteins; TGs: Triglycerides; IMT: Intima media thickness; GFR: Glomerular filtration rate. The data for continuous variables are presented as mean ± SD. The data for categorical variables are presented as percentages. Student *t*-test or Mann-Whitney test were used for comparisons of continuous variables; X^2^ for comparisons of categorical variables: ^a,*^: *p* < 0.05 for the control group *vs.* T2DM + CVD/T2DM − CVD groups; ^a,**^: *p* < 0.001 for the control group *vs.* T2DM + CVD/T2DM − CVD groups; ^b,*^: *p* < 0.05 for comparison between T2DM + CVD *vs.* T2DM − CVD groups; ^b,*^*: *p* < 0.001 for comparison between T2DM + CVD *vs.* T2DM − CVD groups.

### 2.2. Identification of Candidate Biomarkers

Using 2D-DIGE and MALDI TOF/TOF or LC-MS/MS spectrometry methodology, we have identified five spots differentially expressed between groups. The MASCOT database search allowed the protein identification of two spots from MALDI TOF/TOF analysis, and the other three spots were identified by using LC-MS/MS methodology and SEQUEST data analysis program.

The identified spots corresponded to serum retinol binding protein (RBP4), glutathione peroxidase 3 (GPx-3), which were increased, and to transthyretin (TTR), that was decreased in serum of the T2DM patients with CVD compared to the other groups. The chain A of C3b Complement was decreased in the serum of the T2DM patients independently of the presence of CVD compared to the control subjects. The proteins identified by gel trypsin digestion and MALDI-TOF/TOF or LC-MS/MS with detailed information and concentrations compared to the control subjects are summarized in Table 2.

**Figure 1:**
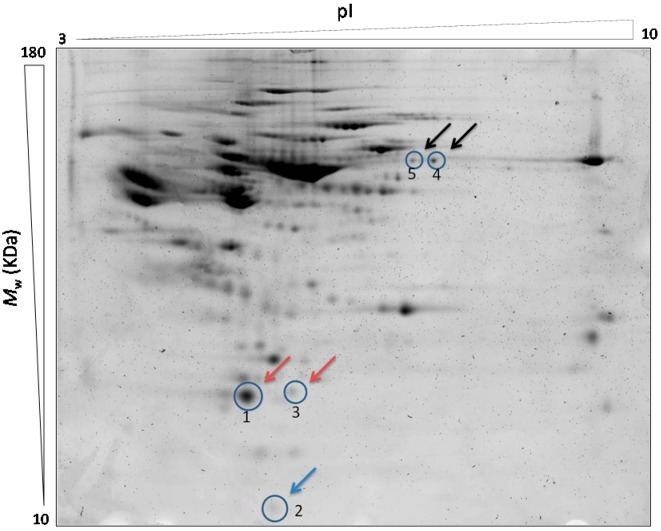
Sypro stained representative 2D-DIGE map of the depleted serum showing the pick location of the proteins differentially expressed. The protein spots found significantly increased (*p* < 0.05) in T2DM + CVD patients compared to the others groups are marked with red arrows; The protein spot found significantly decreased (*p* < 0.05) in T2DM + CVD patients compared to the others groups are marked with blue arrow; The protein spots found significantly decreased in T2DM patients (*p* < 0.05) in regard to the control group are marked with black arrows.

Figure 1 shows a Sypro stained representative map with the pick location of the spots differentially expressed in the serum of the T2DM patients with or without CVD compared to the control subjects.

**Figure 2:**
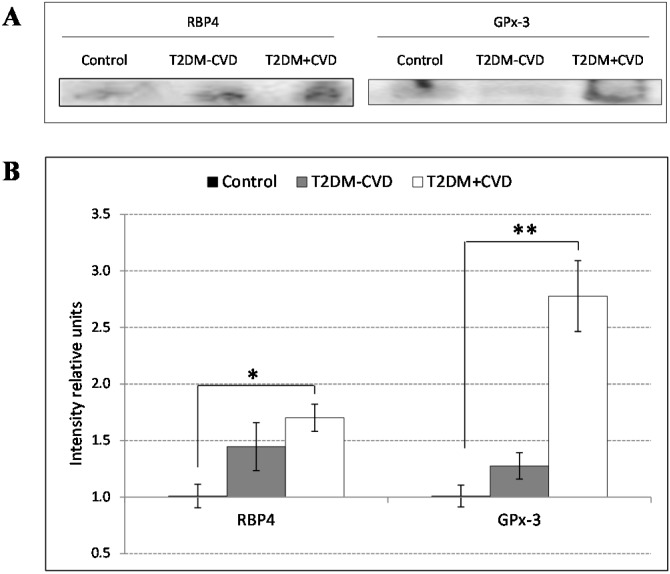
Analysis of RBP4 and GPx-3 expression levels in whole pooled serums from the T2DM patients with and without CVD and the control subjects in three independent experiments of five subjects per group (45 study subjects in total). (**A**) Representative western blot analysis of correspondent RBP4 and GPx-3 pattern showing an increase of RBP4 and GPx-3 in T2DM patients compared to control group; (**B**) Quantification of the protein levels by densitometry analysis of the three western blots showing a significant increase of RBP4 and GPx-3 between T2DM patients with CVD and control subjects. The gel bands were normalized to value 1 corresponding to the control and the protein expression from the T2DM patients with and without CVD is represented relative to the control group. Differences between groups were determined by the Mann-Whitney U test. * *p *< 0.05; ** *p* < 0.001.

In order to confirm the results obtained by 2D-DIGE, we performed a Western blot analysis from 15 whole and pooled serum samples of each group. Thus, in agreement with our results, the spot 1 corresponding to serum RBP4, and the spot 3 correponding to GPx-3, showed significative increased concentrations in the T2DM patients presenting CVD compared to the controls (Figure 2).

However, we were unable to detect TTR and complement C3b in the analysis conducted by western blot.

**Table 2:** List of the candidate biomarkers identified by MALDI-TOF/TOF or LC-MS/MS analysis.

Pos ^a^	Protein Name	ID ^b^	E *M*w/pI ^c^	DeCyder Analysis		MALDI TOF/TOF			LC-MS/MS			Change ^k^
				*p* ^d^	App ^e^	Score ^f^	Sec Cov ^g^	N°pep ^h^	Score ^i^	Sec Cov ^j^	N°pep	
1	Plasma retinol binding protein	Q5VY30	22.5/5.2	0.004	27/27	–	–	–	17.38	12.06	3	↑CVD
2	Transthyretin	P02766	13.8/5.4	0.037	27/27	–	–	–	47.21	68.71	8	↓CVD
3	Gluthathione Peroxidase-3	P22352	22.7/5.3	0.047	24/27	57	96	2	–	–	–	↑CVD
4	Chain A of C3b Complement	P01024	70/7.0	0.012	27/27	495	50	34	–	–	–	↓T2DM
5	Chain A of C3b Complement	P01024	70/6.8	0.023	27/27	310	41	28	–	–	–	↓T2DM

^a^ Position of the spot in 2D-DIGE representative map; ^b^ Protein accession number in Uniprot Database; ^c^ Experimental molecular weight (kDa)/isoelectric point; ^d^ *p *value of the DeCyder analysis according to the ANOVA model; ^e^ Number of the maps in which the spot appears from a total of 27 maps; ^f^ MALDI TOF/TOF protein score; ^g^ Amino acid sequence coverage for the identified protein in percentage; ^h^ number of peptides matched by mass-mass spectrometry; ^i^ LC-MS/MS protein score; ^j^ amino acid sequence coverage for the identified protein in percentage; ^k^ expression change in the T2DM + CVD and T2DM − CVD serum compared to control serum.

## 3. Discussion

Diabetes mellitus currently represents a serious health problem since affects a large proportion of the population [5]. The patients with type 2 diabetes mellitus have increased risk for developing many complications mainly cardiovascular events depicting the major cause of mortality of this disease [6,7]. The probability of developing vascular complications in this population depends on a number of traditional factors and others that are not fully known. The knowledge of new factors involved in these disorders may facilitate the identification of the subjects in whom an intensive approach on cardiovascular risk factors should be established before irreversible damage occurs.

The present study was conducted to analyze the differences in the serum proteome profile of the T2DM patients with and without severe CVD in order to identify the proteins which could be related to higher cardiovascular risk in this population. Since the majority of patients with prolonged duration of T2DM usually show some sign of subclinical vascular disease, we select patients with established cardiovascular disease to identify the proteomic profile associated with more severe and advanced cardiovascular disease.

Our proteomic analysis by using 2D-DIGE and LC-MS/MS revealed changes in proteins related to the inflammatory and redox state. Over recent years, the inflammatory state is becoming more important as a factor involved in the cardiovascular disorders. We found increased RBP4 concentrations in the T2DM patients, being significantly higher in those with CVD compared to those without CVD and the control subjects. RBP4 is a protein, member of the lipocalin family, involved in diverse functions like sensory transduction and carrier of retinol. In plasma, the RBP4-retinol complex interacts with transthyretin, preventing its loss by filtration through the kidney glomeruli. RBP4 is generated by mature adipocytes [8] and activated macrophages [9]. The involvement of RBP4 in insulin resistance has been described in some studies [8,10,11]. Clinical studies have shown that exercise, or other weight loss interventions, could improve the insulin sensitivity by reducing RBP4 serum levels [12,13]. Furthermore, a very recent study determines the mechanism by which RBP4 induces insulin resistance through activation of the antigen presenting cells (APC) of the adipose tissue. This activation induces higher levels of proinflammatory cytokines resulting in an inflammation of the adipose tissue that leads to insulin resistance [14].

However, the association of RBP4 with cardiovascular events and their potential utility as a biomarker of CVD is not well known. In our study we found an increased expression of RBP4 in diabetic patients with cardiovascular disease in regard to the others groups. These findings are consistent with two preliminary studies which show a relationship between RBP4 levels and common cardiovascular risk factors in the elderly population [15,16]. Furthermore, Farjo* et al.* [17] have recently shown that RBP4 promotes the expression of proinflammatory molecules such as vascular cell adhesion molecule 1 (VCAM-1), intercellular adhesion molecule 1 (ICAM-1), *E*-selectin and interleukin 6 (IL-6) through activation of NF-κB and NADPH oxidase in an *in vitro* study. This effect is RBP4-dose dependent and induces the adhesion of leukocytes to the endothelium leading to endothelial inflammation. Our results are in agreement with these recent data suggesting that the elevation of RBP4 could be one of the main causes involved in the development and the progression of CVD associated to T2DM.

Another factor involved in the inflammatory state and vascular lesions is the oxidative stress due to the increase of reactive oxygen species (ROS) [18]. To offset the toxic effects of free radicals produced during vascular lesions over cells, a cascade of redox reactions between nitric oxide (NO), a neurotransmitter with vasodilatory function, and ROS takes place. This process increases lipid peroxidation [19] causing cell damage of the vascular system and aggravating CVD in these patients. Since oxidized lipoproteins constitute one of the most important factors involved in the development of atherosclerosis [20], the endogenous defense systems are essential in the prevention of these complications. According to this, our results revealed an increase of GPx-3 in the serum of diabetes patients with CVD compared to those without CVD and controls. GPx-3 belongs to the family of glutathione peroxidases (GPx’s), a group of enzymes that reduce the oxidative stress decreasing vascular injury. GPx-3 is a homotetrameric protein present in high density lipoproteins (HDL) particles and secreted to the plasma that protects cells and enzymes catalyzing the reduction of hydrogen peroxide, lipid peroxides and organic hydroperoxides. It contributes to maintaining the vascular bioavailability of NO. We postulate that in patients with CVD an increase of GPx-3 levels takes place as a protective mechanism to reduce lipid peroxidation and to maintain the NO levels. There is some evidence supporting this potential protective effect. Regarding this, some studies link increased levels of GPx-3 with vascular complications [21], and a deficiency or a reduction in GPx-3 are related to an increase in the predisposition to developing a thrombotic disorder [22]. Thus, levels of GPx-3 could act as a predictor of cardiovascular risk.

Our results also show a significant decrease in TTR levels in the serum of the T2DM patients presenting CVD as well as a decrease in the chain A of C3 complement levels in the T2DM group compared to the other groups. Although several studies support these findings [23,24], our results about C3 and TTR are not conclusive since these proteins are depleted by the spin cartridges. Thus, results obtained by 2D-DIGE referents to C3 complement and TTR may not reflect the pathophysiological serum levels of patients and controls. So, future studies are needed to clarify the role of these proteins in the development of T2DM and CVD.

The strengths of this study lie in the novel evaluation of the differences in the serum proteome between T2DM patients with and without severe CVD and the identification of the proteins probably involved in the pathogenesis of CVD associated to T2DM and in the strict selection of study subjects. This allows the minimization of confounding factors resulting in a high reproducibility of the results in all replicates. However, our study has certain limitations. A case-control study does not allow us to establish a cause-effect relationship. Moreover, a biochemical validation of the protein candidates by ELISA in a larger T2DM study population will be helpful in order to confirm these preliminary results.

In conclusion, our study shows that proteins involved in the inflammatory state and oxidative stress are associated with cardiovascular complications in patients with type 2 diabetes. The usefulness of these proteins as biomarkers of cardiovascular risk or potential therapeutic targets of vascular disease will require further study.

## 4. Subjects and Methods

### 4.1. Study Population

Our cross-sectional study included 18 males divided according to three study groups: (i) T2DM patients with CVD; (ii) T2DM patients without CVD; (iii) Non-diabetic subjects as controls. To achieve 85.0% statistical power to detect differences among groups it is necessary to include six experimental units per group, resulting in a total sample size of 18 subjects in the experimental study. 

Diagnosis of diabetes was according to the American Diabetes Association criteria (2005). From January 2006 to December 2007 we consecutively recruited patients who had been referred to our outpatient clinic from primary care centers for treatment of diabetes. The inclusion criteria for patients with CVD were coronary heart disease (previous myocardial infarction or coronary revascularization surgery), cerebrovascular disease (ischemic stroke), or ischemic peripheral arterial disease.

The control group consisted of non-diabetic subjects consecutively recruited from the general community in the same period of time and matched with diabetic patients for demographic characteristics.

All were Caucasians, ambulatory and had neither renal, hepatic, gastrointestinal nor thyroid diseases. All T2DM patients were receiving medications for diabetes, including metformin, sulfonylureas, insulin or a combination of these drugs.

Samples of venous blood were obtained from patients in the Endocrinology Unit of University Hospital San Cecilio in Granada. Samples of venous blood in vacutainer tubes containing no anticoagulant were taken in the morning after fasting overnight. Samples were incubated at room temperature for 30–45 min and centrifuged for 15 min at 7500 rpm. Then, the supernatant was carefully aspirated by a serologic pipette, aliquoted into 50 µL cryovials and stored at −80 °C until the examination.

The study was conducted with the approval of the ethical committee of the San Cecilio University Hospital and conformed to the relevant ethical guidelines for human and animal research (Project ID: PI 0514-2012. Research Ethics Committee of Granada Center (CEI-Granada) at 26 November 2012). Written informed consent was obtained from all subjects.

### 4.2. Depletion, Concentration and Cleaning of Serum Samples

A chromatographic removal of the 14 most abundant serum proteins (albumin, IgG, antitrypsin, IgA, transferrin, haptoglobin, fibrinogen, alpha 2-macroglobulin, alpha 1-acid glycoprotein, IgM, apolipoprotein AI, apolipoprotein AII, complement C3 and transthyretin) was performed in 50 µL of whole serum using Multiple Affinity Removal Spin Cartridges (Agilent, Santa Clara, CA, USA) according to the user manual. The depleted fractions were concentrated by a 5 kDa *M*w cut off spin concentrators (Agilent) at 4000 rpm, 60 min at 10 °C. The ReadyPrep 2D cleanup kit (BioRad, Hercules, CA, USA) was used to reduce streaking, background staining, and other gel artifacts that may contaminate the 2D/IEF samples. The resultant pellet was dispersed into the adequate volume of 2D gel electrophoresis buffer (8 M Urea, 4% CHAPS) to solubilize protein samples. The samples were separated in aliquots of 11.5 µL for DIGE assay and 0.5 µL for protein determination, pH adjustment and 1D electrophoresis.

### 4.3. 2D-DIGE

#### 4.3.1. CyDye Fluors Preparation and Sample Labeling

The CyDye DIGE fluors (Kit labeling minimal CyDye DIGE fluor 1 × 5 nmol (GE Healthcare, Freiburg, Germany)) were dissolved in dimethylformamide up to 400 pmol/mL of working solution.

Sodium hydroxide at 0.1 M diluted 1:10 was added to each sample to adjust pH at 8.5–9 for the labeling reaction. Randomization of samples across gels removes any bias from the experiments. Fifty µg of protein from each sample was minimally labeled on ice for 30 min with 1 µL of corresponding fluorescent dye working solution according to the Ettan DIGE System user manual (GE Healthcare). The internal standard (IS) was generated by combining equal amounts of each sample and labeled with Cy2 fluorescent dye. The reaction was stopped by addition and incubation of 10 mM lysine for 10 min on ice in the dark (1 µL Lys per 50 µg protein). To prepare the samples for each gel, two samples were mixed with 50 µg of IS according to the user manual of 2D-DIGE GE Healthcare (Table 3). Twenty mM DTT and 0.5 mM PMSF were added to the mix. Finally, a total of nine 2D gels were run.

**Table 3:** Sample arrangement on each gel and staining with each corresponding fluorophore. A: T2DM + CVD group; B: T2DM − CVD group; IS: Internal standard.

Gel Number	Cy2	Cy3	Cy5
**1**	IS	Control 1	Sample B4
**2**	IS	Sample A1	Control 4
**3**	IS	Sample B1	Sample A4
**4**	IS	Control 2	Sample B5
**5**	IS	Sample A2	Control 5
**6**	IS	Sample B2	Sample A5
**7**	IS	Control 3	Sample B6
**8**	IS	Sample A3	Control 6
**9**	IS	Sample B3	Sample A6

#### 4.3.2. 2D Gel Electrophoresis

IPG (0.5%) buffer was added to destreak solution (GE Healthcare) after 30 min to temper the solution at RT. The adequate volume was added to the sample and placed into the strip holders. The IPG strips (pH 3–10, 24 cm, GE Healthcare) were located over the sample, covered with mineral oil and focused on an IPGphor III (GE Healthcare) following these steps: 1 h at 500 V, 1 h at 1000 V, 3 h at 8000 V, 8000 V until 20,000 V/h and 500 V as maintenance step. The run was monitorized by IPGphor III software (GE Healthcare) ensuring that isoelectric focusing (IEF) process was successful. The strips were twice equilibrated for 15 min in the adequate buffer containing 75 mM Tris buffer (pH 8.8), 6 M Urea, 30% glycerol, 2% SDS and Bromophenol Blue. The first equilibration was with 1% DTT, and the second one with 2.5% Iodoacetamide in the equilibration buffer. The second dimension was carried out using 12% polyacrylamide gels at 13 mA per gel for 1 h in the first step and 17 mA per gel for 5.50 h in the second step. The proteins were visualized by Typhoon 9400 fluorescence scanner (GE Healthcare). The image analysis was performed by the specific software DeCyder 7.0 (GE Healthcare), that performs an automatic analysis and a co-detection of the fluorescent gels images by background subtraction, normalization, inter-gel matching and quantization. By using the statistical analysis by Decyder 7.0 (GE Healthcare) the sum of the pixel values within a spot minus background for each experimental sample is compared directly to the internal standard. Thereby, the protein abundance for each spot is expressed as a normalized ratio relative to the internal standard. This analysis compares the average ratio and variation within each group to the average ratio and variation in the other groups to see if any change between the groups is significant. Thus, it is possible to compare the protein abundance for a protein of interest in different samples. The total proteins were detected by Sypro staining (BioRad).

#### 4.3.3. Identification of the Candidate Biomarker by MALDI-TOF/TOF

The analysis by Decyder 7.0 performs the matching of the gels and creates a pick list after the scan. Selected protein spots are automatically picked from 2D representative gel by Ettan Spot Picker (GE Healthcare) using the pick list generated. The gel plugs are transferred into microplates for further digestion prior to identification.

The gel pieces were reduced with DTT for 60 min at room temperature and cysteines were carbamidomethylated with iodoacetamide for 30 min. The proteins were digested with trypsin (Gold, MS Grade, Promega, Madison, WI, USA) for 8 h at 37 °C. The resulting peptide mixture was spotted on a MALDI plate with CHCA and analyzed using the 4800 MALDI TOF/TOF mass spectrometer (AB-Sciex, Madrid, Spain). The MALDI-TOF/TOF spectra were interpreted by database search MASCOT (Matrix Science, Boston, MA, USA) with a significance threshold of the MOWSE score of *p* < 0.05. The database used for identification was NCBI restricted to mammalian proteins with the following parameters: peptide mass tolerance 100 ppm, fragment tolerance 0.2 Da, enzyme set as trypsin and allowance up to one missed cleavage, variable modification of methionine oxidation (+16 Da), fixed modification of cysteine carbamidomethylation (+57 Da). Only the proteins with the better identification parameters and the best score during the search in databases have been included in the list of results. Other identified proteins with lower score were identified (Data not shown). 

#### 4.3.4. LC-MS/MS Analysis

The tryptic extracts were reanalyzed by high resolution LC-MS/MS in data-dependent mode. The MS system used was an Orbitrap XL (ThermoFisher, Waltham, MA, USA) equipped with a microESI ion source (Proxeon). The tryptic extracts were diluted to 20 µL with 5% methanol and 1% formic acid. Then, extracts were loaded into a chromatographic system consisting of a C18 preconcentration cartridge (Agilent, Santa Clara, CA, USA) connected to a 15 cm long, 100 µm i.d. C18 column (Nikkyo Technos Co., Tokyo, Japan). The separation was done at 0.4 µL/min in a 30-min acetonitrile gradient from 3% to 40% (solvent A: 0.1% formic acid, solvent B: acetonitrile 0.1% formic acid). The HPLC system was composed of a 1200 capillary nano pump (Agilent), a binary pump, a thermostated micro injector and a micro switch valve. The Orbitrap XL was operated in the positive ion mode with a spray voltage of 2 kV. The scan range for full scans was *m*/*z* 400–2000. The spectrometric analysis was performed in a data-dependent mode, acquiring a full scan followed by 10 MS/MS scans of the 10 most intense signals detected in the MS scan. An exclusion time of 30 s was included to avoid repetitive MS/MS analysis of the dominant MS signals. LC-MS/MS spectra were searched using SEQUEST (Proteome Discoverer v1.3, ThermoFisher) with the following parameters: peptide mass tolerance 10 ppm, fragment tolerance 0.8 Da, enzyme set as trypsin and allowance up to two missed cleavages, dynamic modification of methionine oxidation (+16 Da), fixed modification of cysteine carbamidomethylation (+57 Da).

The database used for searching included human proteins (Uniprot taxonomy_9606). The peptide identifications were filtered for 0.1% FDR and only proteins identified with two or more peptides were considered. Only the proteins with the better identification parameters and the best score during the search in databases have been included in the list of results. Other proteins with lower score were identified (Data not shown).

#### 4.3.5. Western Blot Analysis

A biochemical validation of protein candidates was performed by western blot analysis using standard techniques. In order to get a better approximation to the physiologic state of the individuals we used whole serum samples. We performed the division of 15 samples from each study group into 3 groups of 5 individuals each one, giving a total of 9 groups (3 groups of Control, 3 groups of T2DM + CVD and 3 groups of T2DM − CVD). The 9 groups were compared in three independent Western blots. Equal volumes of the pooled samples were separated on a 1D SDS-PAGE using 15% polyacrylamide gels during 60 min at 150 V. The separated proteins were transferred to PVDF membrane on Trans-Blot Turb Transfer System (BioRad), following the transfer protocol for 1.5 mm gels. The membranes were blocked in phosphate-buffered saline (PBS) supplemented with 5% (*w*/*v*) non-fat dry milk (BioRad) for 2 h at RT. Afterwards the membranes were washed with PBS 0.05% Tween 20 (PBST) during 10 min and subsequently they were incubated overnight with polyclonal human anti-goat primary antibodies: Anti-serum retinol binding protein (RBP4) ((SAB2500875), Anti-Transthyretin (TTR) (SAB25001931), Anti-glutathione peroxidase 3 (GPx-3) (SAB2500485) Sigma-Aldrich, Madrid, Spain); Anti-C3 complement ((A304) Quidel, San Diego, CA, USA), at 4 °C in PBS containing 0.1% albumin (PBSA). After three washes with PBST, the membranes were incubated with corresponding secondary horseradish peroxidase-conjugated antibody (A5420, Sigma-Aldrich) diluted 1:20,000 in PBSA for 1 h at RT. The proteins were detected by chemiluminiscence using Clarity Western ECL Substrate (BioRad) and the ChemiDoc gel imaging system (BioRad). The bands were quantified in three independent experiments by Quantity One software (BioRad), normalized to value 1 corresponding to the control and expressed as intensity difference relative to the control group (ratio disease group/control group).

### 4.4. Statistical Analysis

The data for continuous variables are presented as mean ± standard deviation (SD). The data for categorical variables are presented as numbers and/or percentages. Kolmogorov-Smirnov test was used to test the normal distribution of continuous variables. Comparisons of categorical variables among groups were performed using the Chi-square test. Comparison of continuous variables between case-control was performed using the unpaired Student’s *t* test, or the Mann-Whitney test. The statistical significance was set at *p* < 0.05 (two-tailed) and performed with the specific software, SPSS 18.0 (Chicago, IL, USA).

For statistical analysis by Decyder 7.0, the individual gels were processed in Differential In-gel Analysis module (DIA) to spot detection and first in-gel analysis for the images from each single gel removing background and artefacts and normalizing the gel images. The Biological Variation Analysis module (BVA) was used to analyze DIA spot maps of all gels in one DIGE experiment, to merge gel spot maps, to normalize intensities and to obtain statistical differences between groups. The analysis of variance (ANOVA) was applied to matched spots. The data was filtered to retain spots with ANOVA *p*-values of 0.05 or less.

To get a power of 85% to find differences in testing the null hypothesis “The proportions of the three groups are equal using a Chi-square test for three independent samples, considering that the significance level is 5% and assuming that the expected proportions of the three groups are specified by the researcher (85%, 10%, 10%)”, it is necessary to include 18 experimental units distributed equally among the 3 groups (6 units per group).

## References

[1] Jensen M.K., Bertoia M.L., Cahill L.E., Agarwal I., Rimm E.B., Mukamal K.J. (2014). Novel metabolic biomarkers of cardiovascular disease. Nat. Rev. Endocrinol..

[2] Tousoulis D., Papageorgiou N., Androulakis E., Siasos G., Latsios G., Tentolouris K., Stefanadis C. (2013). Diabetes mellitus-associated vascular impairment: Novel circulating biomarkers and therapeutic approaches. J. Am. Coll. Cardiol..

[3] Paneni F., Costantino S., Cosentino F. (2015). Role of oxidative stress in endothelial insulin resistance. World J. Diabetes.

[4] Zhang R., Barker L., Pinchev D., Marshall J., Rasamoelisolo M., Smith C., Kupchak P., Kireeva I., Ingratta L., Jackowski G. (2004). Mining biomarkers in human sera using proteomic tools. Proteomics.

[5] Chen L., Magliano D.J., Zimmet P.Z. (2012). The worldwide epidemiology of type 2 diabetes mellitus—Present and future perspectives. Nat. Rev. Endocrinol..

[6] Fruchart J.-C., Davignon J., Hermans M.P., Al-Rubeaan K., Amarenco P., Assmann G., Barter P., Betteridge J., Bruckert E., Cuevas A. (2014). Residual macrovascular risk in 2013: What have we learned?. Cardiovasc. Diabetol..

[7] Iglesias P., Pedro-Botet J., Arrieta F., Aguilar M., Escobar F. Clinical significance of the new cardiovascular risk markers in diabetes mellitus. Curr. Diabetes Rev..

[8] Friebe D., Neef M., Erbs S., Dittrich K., Kratzsch J., Kovacs P., Blüher M., Kiess W., Körner A. (2011). Retinol binding protein 4 (RBP4) is primarily associated with adipose tissue mass in children. Int. J. Pediatr. Obes..

[9] Broch M., Ramírez R., Auguet M.T., Alcaide M.J., Aguilar C., Garcia-Espana A., Richart C. (2010). Macrophages are novel sites of expression and regulation of retinol binding protein-4 (RBP4). Physiol. Res..

[10] Shaker O., El-Shehaby A., Zakaria A., Mostafa N., Talaat S., Katsiki N., Mikhailidis D.P. (2011). Plasma visfatin and retinol binding protein-4 levels in patients with type 2 diabetes mellitus and their relationship to adiposity and fatty liver. Clin. Biochem..

[11] Park S.E., Lee N.S., Park J.W., Rhee E.-J., Lee W.-Y., Oh K.-W., Park S.-W., Park C.-Y., Youn B.-S. (2014). Association of urinary RBP4 with insulin resistance, inflammation, and microalbuminuria. Eur. J. Endocrinol..

[12] Balagopal P., Graham T.E., Kahn B.B., Altomare A., Funanage V., George D. (2007). Reduction of elevated serum retinol binding protein in obese children by lifestyle intervention: Association with subclinical inflammation. J. Clin. Endocrinol. Metab..

[13] Haider D.G., Schindler K., Prager G., Bohdjalian A., Luger A., Wolzt M., Ludvik B. (2007). Serum retinol-binding protein 4 is reduced after weight loss in morbidly obese subjects. J. Clin. Endocrinol. Metab..

[14] Moraes-Vieira P.M., Yore M.M., Dwyer P.M., Syed I., Aryal P., Kahn B.B. (2014). RBP4 activates antigen-presenting cells, leading to adipose tissue inflammation and systemic insulin resistance. Cell Metab..

[15] Sasaki M., Otani T., Kawakami M., Ishikawa S.-E. (2010). Elevation of plasma retinol-binding protein 4 and reduction of plasma adiponectin in subjects with cerebral infarction. Metabolism.

[16] Ingelsson E., Sundström J., Melhus H., Michaëlsson K., Berne C., Vasan R.S., Risérus U., Blomhoff R., Lind L., Arnlöv J. (2009). Circulating retinol-binding protein 4, cardiovascular risk factors and prevalent cardiovascular disease in elderly. Atherosclerosis.

[17] Farjo K.M., Farjo R.A., Halsey S., Moiseyev G., Ma J.-X. (2012). Retinol-binding protein 4 induces inflammation in human endothelial cells by an NADPH oxidase- and nuclear factor κ B-dependent and retinol-independent mechanism. Mol. Cell. Biol..

[18] Abe J.-I., Manabe I., Aikawa M., Aikawa E. (2013). Cardiovascular inflammation 2012: Reactive oxygen species, SUMOylation, and biomarkers in cardiovascular inflammation. Int. J. Inflamm..

[19] Beckman J.S., Beckman T.W., Chen J., Marshall P.A., Freeman B.A. (1990). Apparent hydroxyl radical production by peroxynitrite: Implications for endothelial injury from nitric oxide and superoxide. Proc. Natl. Acad. Sci. USA.

[20] Ehara S., Ueda M., Naruko T., Haze K., Itoh A., Otsuka M., Komatsu R., Matsuo T., Itabe H., Takano T. (2001). Elevated levels of oxidized low density lipoprotein show a positive relationship with the severity of acute coronary syndromes. Circulation.

[21] Russo C., Olivieri O., Girelli D., Faccini G., Zenari M.L., Lombardi S., Corrocher R. (1998). Anti-oxidant status and lipid peroxidation in patients with essential hypertension. J. Hypertens..

[22] Jin R.C., Mahoney C.E., Coleman Anderson L., Ottaviano F., Croce K., Leopold J.A., Zhang Y.-Y., Tang S.-S., Handy D.E., Loscalzo J. (2011). Glutathione peroxidase-3 deficiency promotes platelet-dependent thrombosis *in vivo*. Circulation.

[23] Ingenbleek Y., Young V. (1994). Transthyretin (prealbumin) in health and disease: Nutritional implications. Annu. Rev. Nutr..

[24] Lappas M. (2011). Lower circulating levels of complement split proteins C3a and C4a in maternal plasma of women with gestational diabetes mellitus. Diabet. Med..

